# Two new doping-free manufacturing processes for bread-derived carbon electrodes with control over micro- and macro-topological surface features

**DOI:** 10.1098/rsos.240953

**Published:** 2025-02-19

**Authors:** David Bujdos, Zachary Kuzel, Adam Wood

**Affiliations:** ^1^ Department of Engineering, Saint Vincent College, Latrobe, PA 15650, USA; ^2^ Department of Industrial Engineering, University of Pittsburgh, Pittsburgh, PA 15261, USA

**Keywords:** pyrolysis, stamping, reconstituting, carbon electrode, electrode precursor

## Abstract

Pyrolyzed carbon electrodes (PCEs) can provide sustainable alternatives in electric devices, but it is difficult to control their surface geometries during their semi-destructive fabrication procedure. Impressive contributions have been made to the field of PCE fabrication in terms of the nanoscale, functionalization, and separation applications; however, further progress towards an emphasis on a sustainable life cycle is the next step forward. Here, we propose two new methodologies for creating sustainable PCEs: stamping, where a user-designed, 3D printed electrode precursor (EP) imparts a shape on an organic material, and reconstitution, where the same EP acts as a mould as a mixture of agitated organic material and water dries to leave behind a rigid shape. Both methods allow for the reuse of the EP and the upcycling of biologically derived waste products as a pyrolytic input, and they do not require chemical modification. A comparison of the two methodologies is discussed as surface features of PCEs scale by a factor of 0.78 during the reconstitution process and by a factor of 0.68 during the stamping process. These PCEs maintain defined structures on the micro-scale and demonstrate previously unachievable resolution to the naked eye prior to these two novel pathways.

## 1. Background

Most commercially available electronic devices are composed of metallic materials due to their excellent conductive properties; however, these components can account for up to 60% of discarded electronic equipment such as DVD players, television sets and cell phones [1]. Furthermore, metals such as cadmium, copper, and zinc are also wasted through the demolition of construction sites and can pose environmental risks in high concentrations [2]. Steps have been taken to sort through such metallic waste to mitigate these effects and recycle viable components, but this process can be over 10 steps at times [3]. Even before the recycling stage of the life cycle analysis of these metals, researchers have questioned the safety of ongoing mining operations in certain parts of the world. Harvesting and mining can expose toxic quantities of heavy metals to humans and the environment [4]. Thus, the discovery of an alternative metallic material would be beneficial from an environmental, industrial, and safety-oriented perspective. Recent advances in sustainable electronics and the advent of wearable technology such as smart watches, smart glasses, and wireless audio entertainment all advocate for graphene and carbon-based composite as one such alternative material. Often considered the ‘most esteemed’ material, graphene and graphene oxide nanocomposites have a wide range of applications in energy storage, heat transfer, structural stability, hierarchical materials, soft matter, and electrical conductivity [5,6].

Pathways to graphene-like materials have been of interest due to graphene’s attractive properties. In some cases, the ability to harvest carbon structures from naturally occurring materials could yield graphitic substances in a laboratory setting without requiring expensive reactants. The pathway of focus for this study is pyrolysis, a type of controlled anaerobic decomposition of organic material at high temperatures [7,8]. Pyrolysis can be used to expel unwanted macro-molecules via continuous purging with inert gas and produce a solid, monolithic, carbon-rich surface. Several biologically sourced inputs such as beans, leaves, and date shells can be pyrolyzed to reduce the composition of the biomaterial down to its carbon backbone, similar to the structure of graphene [9–11]. After decomposition at high temperatures, the resulting material is electrically conductive. In some circumstances, these PCEs can even be functionalized from waste bread to applications of desalination to give a new perspective on the idea of a sustainable life cycle [12]. Furthermore, literature has already been provided to examine the role of pyrolysis in industrial waste management to generate high energy-density carbon derivatives [13,14]. As such, pyrolysis will serve as the primary method for the conversion of waste biomaterial to PCE for this study.

## 2. Introduction

The pyrolysis of organic materials has been investigated thoroughly as a method to provide an alternative conductive material, producing a wide range of applications and manufacturing techniques. The list of available organic materials can even be broadened to several walks of life including plants, animals, and fungi, though additional physical and/or chemical treatments are often required to prepare such materials for use in pyrolytic carbon systems [15–18]. In addition to the thermal deformation process, a common procedure called doping is used to combine chemical additives to enhance the desirable properties of the final PCE. Some doping agents in PCEs can include nitrogenous species, metallic atoms, or even a combination of both. Altering the ratio of doping agent to organic material can influence the electrical properties of the resulting PCE and can even introduce the ability to detect halogen ions in aqueous media [19–21]. Doping can also impact the chemical performance of the carbon-rich material and can be used to increase the functionality of free-standing carbon electrodes for desalination purposes [22,23]. Doping the surface of pre-pyrolytic material with acid can lead to an exfoliated nanosheet structure with excellent ion transport capabilities or a graphene oxide structure for capacitor applications when paired with various electrolytes [24,25]. Another study leverages metallic doping of a species of pyrolyzed leaves to create graphene nanosheets with improved metallic character; the resulting power-dense material can be used in capacitive systems with polymer electrolytes. Still, maintaining an eco-friendly fabrication procedure for the metal-doped material is described as a future challenge [26]. Thus, the process of doping has produced beneficial results regarding the performance of pyrolyzed electrodes, but it can be costly and unsustainable to incorporate complex chemical compounds into the PCE structure.

In addition to the cost of doping agents, several other processing techniques for PCEs have achieved impressive levels of performance but require a sophisticated or expensive manufacturing method. Investigations on the technoeconomic viability of such systems that may employ PCEs have raised questions about the role of cost in production, so close attention should be paid to the fiscal impact of future PCE systems [27]. One common method of PCE production involves directly 3D printing the material that will eventually compose the PCE. The use of 3D printing technologies allows for rapid prototyping and the ability to incorporate higher levels of dimensional control, which can make PCE fabrication more precise [28]. Since 3D printing often involves a polymeric material, the resulting print is rich in carbon as most polymers are comprised of carbon chains. The direct pyrolysis of a 3D printed object can yield high-resolution PCEs with beautiful detail and tight control over feature size [29]. Several teams have demonstrated the effectiveness of directly 3D printing an electrode with a carbon-based material and using pyrolysis to initiate the beneficial conductive properties of these direct carbon electrodes. Highly detailed and architected structures, neurotransmitter sensors, and flexible capacitors have all been fabricated using 3D printing as a pathway to PCE [30–33]. While these achievements are remarkable, the direct pyrolysis of a 3D printed object requires repeated printing for each PCE, reducing its cost-effectiveness at an industrial-scale level of production.

**Figure 1.**
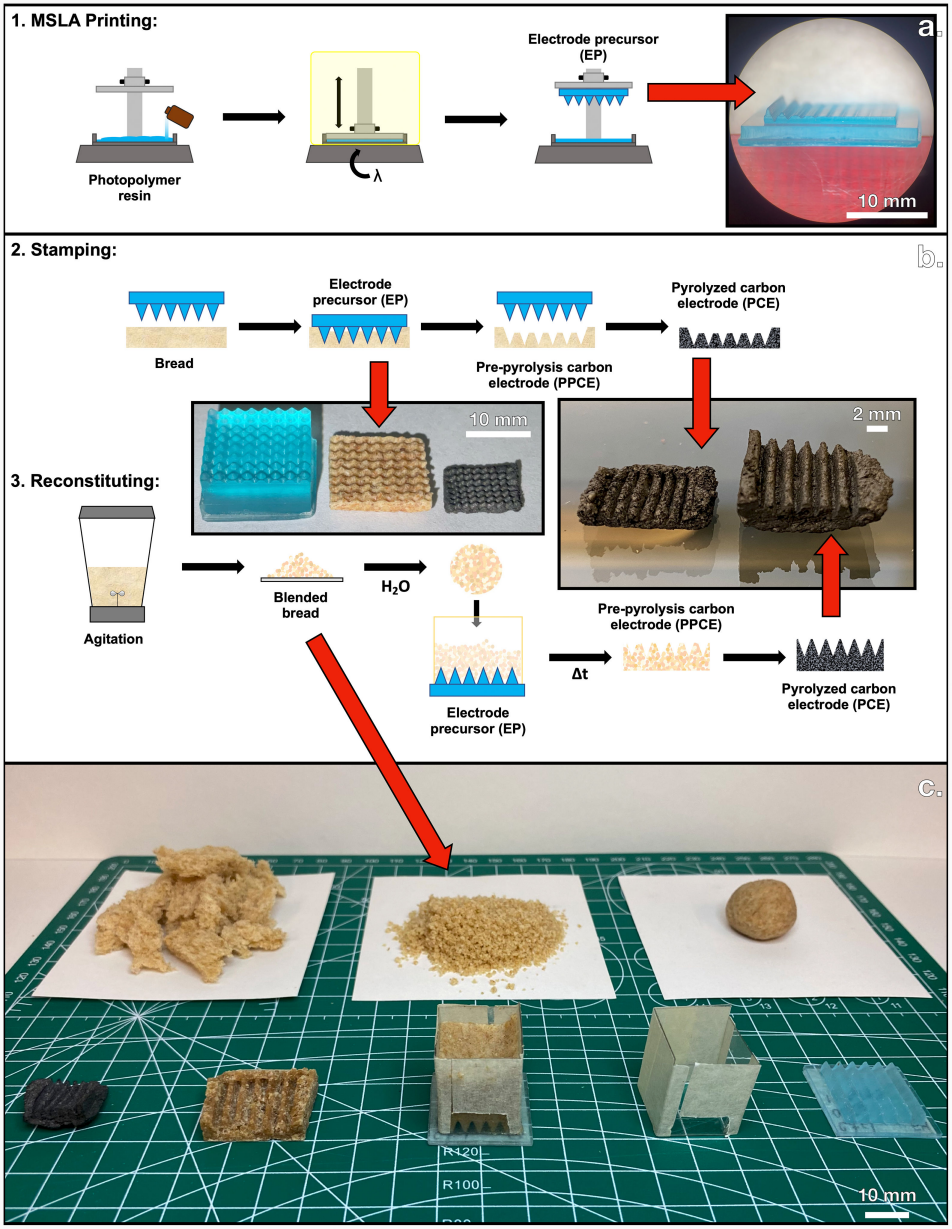
Schematic of the stamping and reconstituting manufacturing processes.

Carbon electrodes produced through pyrolysis are not subject to the same manufacturing techniques that the ductility and malleability of metals afford. If carbon electrodes can serve as a viable alternative, then a manufacturing method that supports the nanoscale, microscale, and macroscale should be discovered. Teams from research institutions have already uncovered levels of nanoscale and microscale control of carbon electrodes and have even demonstrated the possibility of using biologically sourced materials as an input [34–36]. Attempts at the macroscale have likewise shown promising results but make use of 3D printing technology or doping agents to achieve detailed origami-like designs [37–39]. Control of the final structure from microporous to mesoporous has also been achieved through the variation of pyrolysis temperature of acid-doped PCEs [40]. Future manufacturing techniques for viable and sustainable PCEs must (i) be free of doping agents, (ii) adhere to renewable and cheap pathways of production, and (iii) explore the possibility of macroscale design for industrial scalability. This work aims to answer these needs by proposing two sustainable manufacturing processes that use no doping or direct 3D printing/reprinting of pre-pyrolysis material. These two approaches are outlined with a graphical overview in figure 1.

## 3. Methods

**Figure 2.**
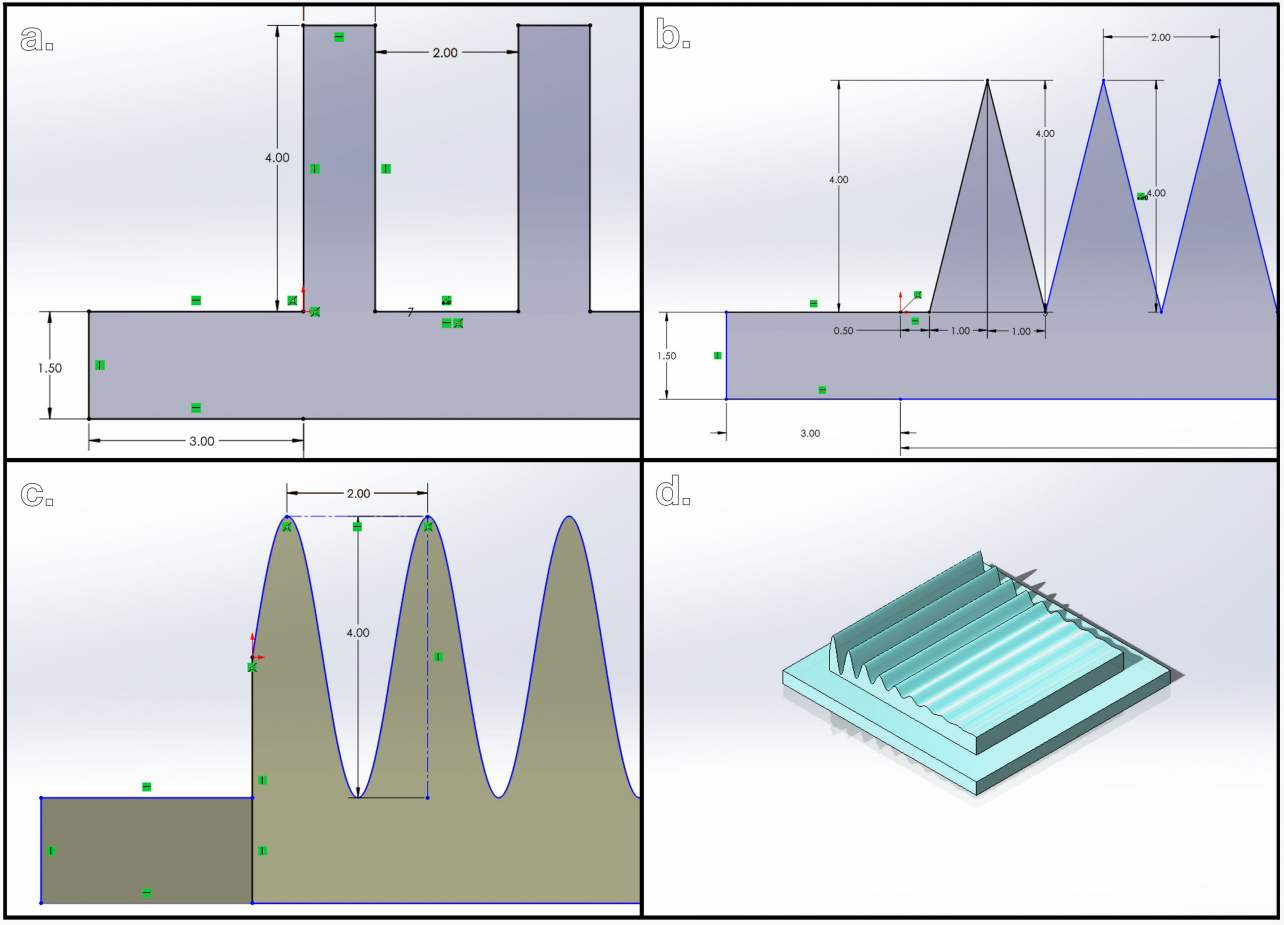
(*a*) Square wave electrode precursor (EP). (*b*) Saw wave EP. (*c*) Sine wave EP. (*d*) Three-dimensional rendering of a function-driven EP.


Though 3D printing will not be employed here to provide the direct material for pyrolysis, masked stereolithography (MSLA) 3D printing can allow for feature design without destroying the 3D printed material in pyrolysis [41]. Standard MSLA printing techniques using photoactivated resin and additive manufacturing provide the pathway for creating EPs by sequentially curing layers of resin with UV radiation [42]. A schematic of the MSLA printing process for generating EPs is shown in figure 1*a*. Using computer-aided design software, the user can tune the dimensions of EPs to any desired shape of the final PCE. Figure 2*a*–*c* shows side profiles of three example EP shapes rendered using SOLIDWORKS. In this work, three different shapes of EPs were studied to rule out the effect of shape on final feature retention. Namely, these EP shapes are a square wave (figure 2*a*), saw wave (figure 2*b*) and sine wave (figure 2*c*); the last of the three shapes takes advantage of the function-driven curve feature in SOLIDWORKS and is mapped using equation (3.1).

(3.1) y = 2sin⁡(πx),  [0≤x≤19]

**Figure 3.**
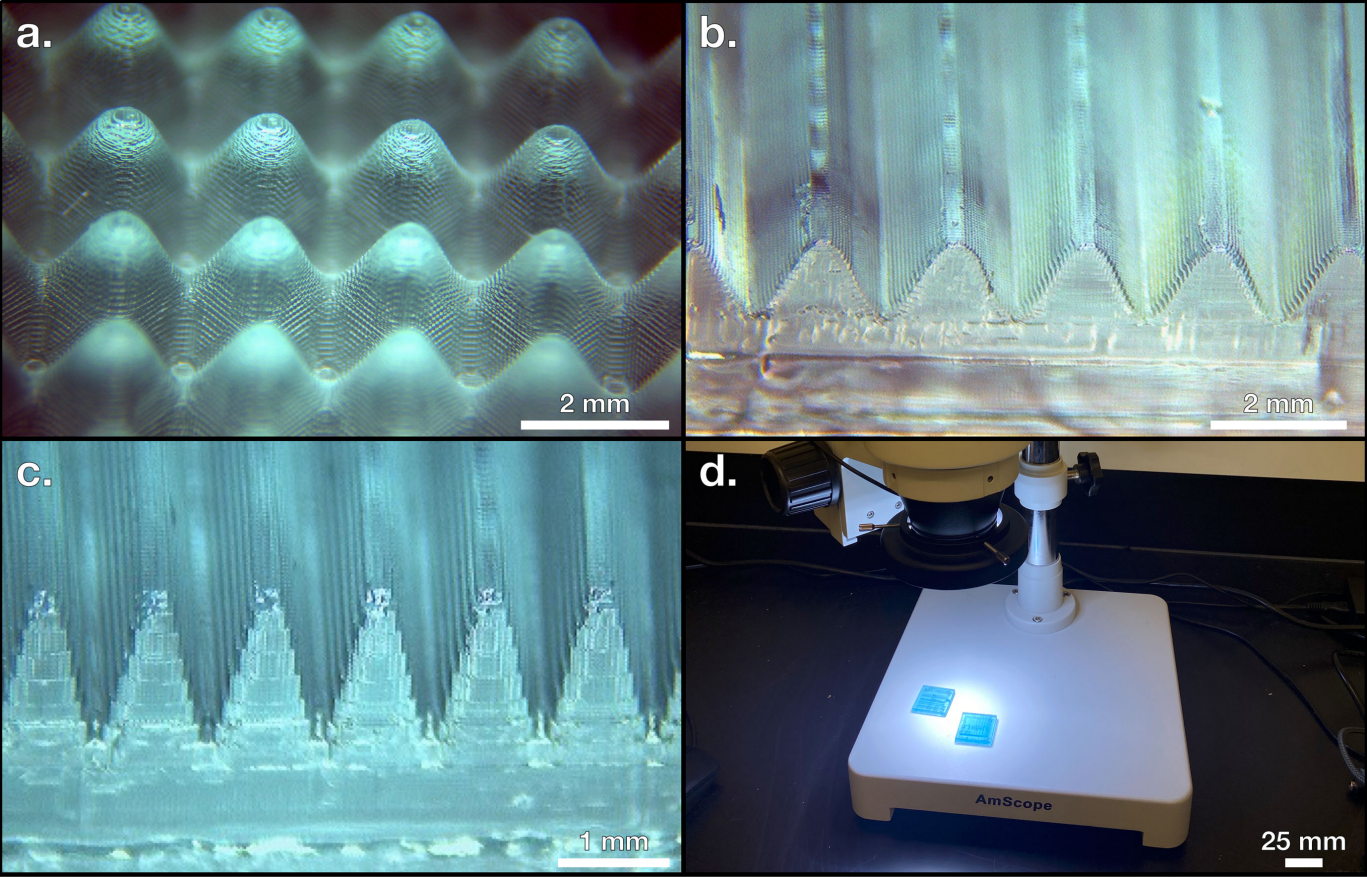
(*a*) Sine matrix electrode precursor (EP). (*b*) Sine wave EP. (*c*) Sine wave EP with narrower features. (*d*) Perspective view shown with microscope for scale.

**Figure 4.**
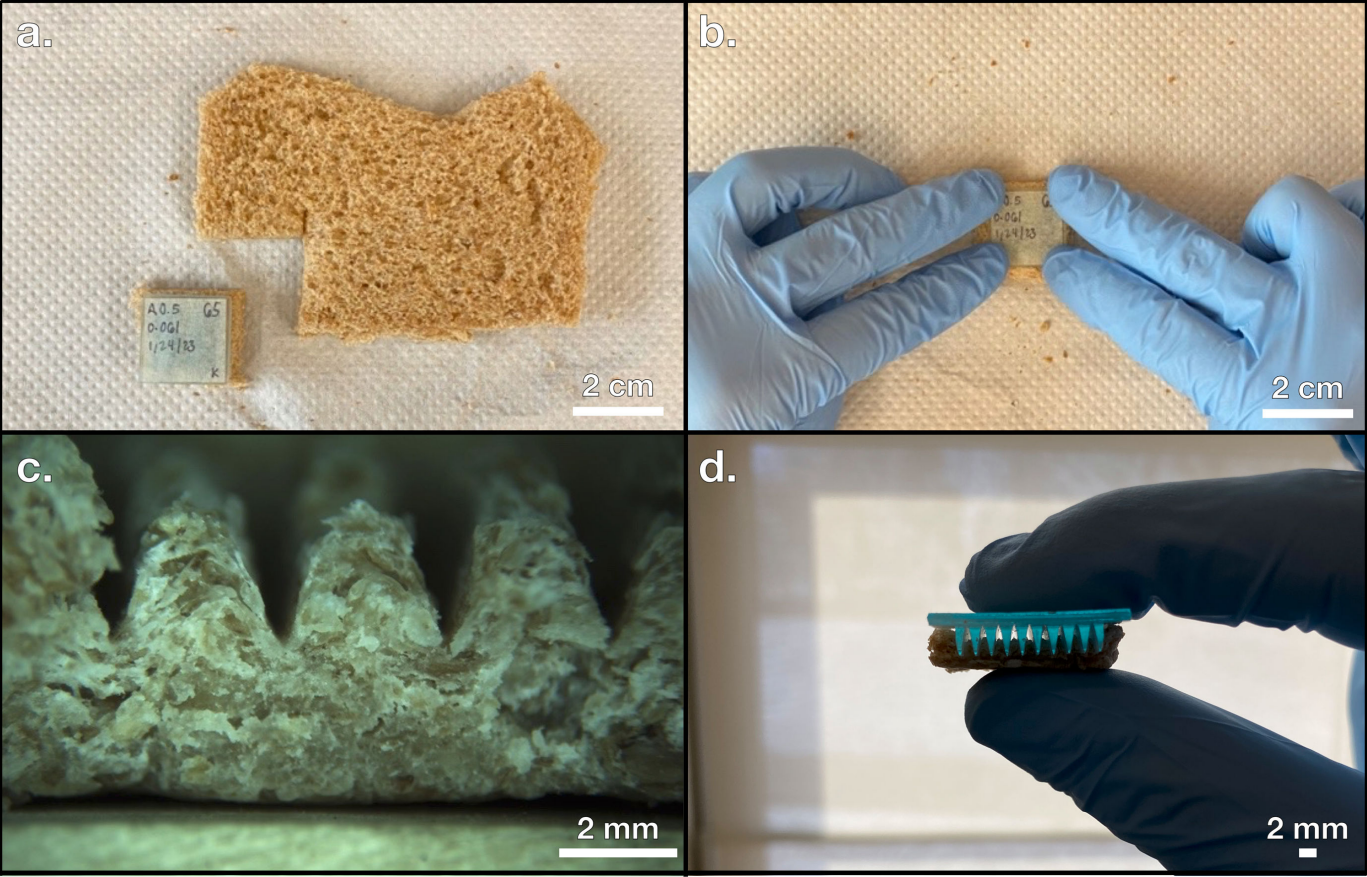
(*a*) Bread prepared for stamping. (*b*) Imparting the electrode precursor (EP) features onto the bread with force. (*c*) Pre-pyrolyzed carbon electrode (PPCE). (*d*) Incomplete surface area contact during stamping.

The key advantage of using pyrolytically indirect 3D printing techniques is that any one EP acts as a mould; the number of PCEs created from one EP is essentially limitless. With pyrolytically indirect 3D printing, only a single EP needs to be fabricated using a 3D printer. After a parent EP is created, the pattern of the EP may be imparted to as many potential electrodes as the feedstock will allow since the EP itself is removed prior to pyrolysis. After printing a variety of EPs using a Photon Mono X and a Photon Mono X 6 K resin printer, representative samples were photographed in an AmScope LED-144 stereo microscope using a Moticam 5.0 MP digital camera, shown in figure 3. In addition to securing a reusable method of creating EPs, a source of pre-pyrolytic material must be chosen to provide the monolithic structure of the PCE. Keeping in mind the importance of a sustainable production pathway, waste bread is reported to be discarded at a high rate and can serve as an abundant and cheap reactant [43]. The broad range of biomaterials available for pyrolysis has already been explored [15], and since bread is a dry, low-density, humane, and readily available input, a loaf of Pepperidge Farms 100% whole wheat sliced bread is selected as the organic material to generate PCEs in a commitment to sustainable sourcing of renewable feedstocks [44]. This type of bread is well-suited for this work since it is widely commercially available and has been previously studied as a source for PCEs [12]. To indirectly impart the user-defined shape to the bread without spending the 3D printed EP, the EPs were physically forced into the surface of the bread to achieve shape retention in a few seconds as shown in figure 4*b*. This procedure of forcing the desired surface of the EP onto the surface of the bread is referred to as stamping and allows for the translation of two-dimensional and some three-dimensional features (without overhang) into pre-pyrolyzed carbon electrodes (PPCEs). An overview of the stamping process is shown in figure 1*b* and the results of stamping an EP onto a PPCE are shown in figure 4*c*,*d*.

While the stamping process takes less than a minute to produce a PPCE, the primary challenge of this procedure is achieving full pattern transfer from EP to PPCE. Notice that in figure 4*d*, it is clear that an EP with deep surface features often does not reach the surface of the bread to the point where full surface area contact is achieved. Part of the deep-lying EP surface highlighted by the backlit area in the troughs between features does not impart a shape. To quantify this relationship directly, a variable named the aspect ratio is defined in equations (3.2) and (3.3) for the three wave-like EP geometries shown in figure 2.


(3.2) $$R=\frac{h}{\omega }$$

(3.3) h=2A

where *R* represents the aspect ratio, a dimensionless parameter, *A* represents the amplitude of the wave measured in mm, *h* represents the total vertical feature distance of the EP measured in mm and *ω* represents the horizontal distance between EP peaks measured in mm. The EP in figure 4*d* has an aspect ratio of 2, so a new method of producing a PPCE from an EP needs to be determined that can achieve complete surface area contact for EP features with an aspect ratio of 2.

**Figure 5.**
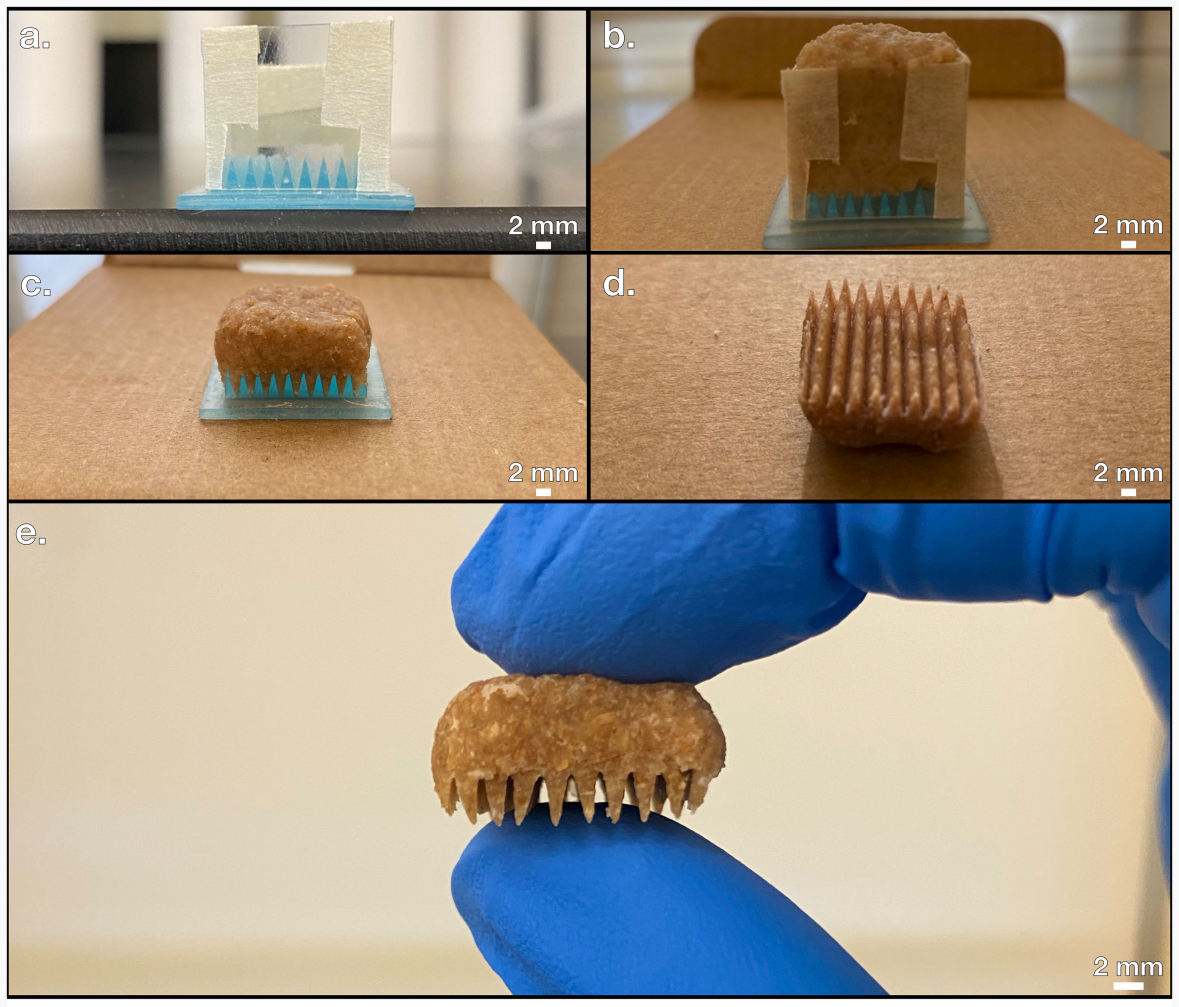
(*a*) Plastic scaffolding for electrode precursors (EPs). (*b*) Drying stage 1. (*c*) Drying stage 2. (*d*) Drying stage 3. (*e*) Complete pre-pyrolyzed carbon electrode (PPCE) made with the reconstitution process.

To solve this problem, another method is proposed here called reconstituting. An overview of this process is shown in figure 1*b*,*c*. The same EPs generated with MSLA 3D printing described in the previous section are reused here; instead of acting as stamps to impress a pattern onto the surface of bread with force, the EP is used as a mould to cast its features on a soft mixture of bread and water. To achieve the reconstituted phase, the bread was first agitated in a Mainstays 6-Speed blender to destroy the inherent architecture present in the bread from leavening. This reduces the bread’s resistance to normal stress and allows for the features of the EP to penetrate deeper into the surface of the bread. After blending, the sand-like bread powder was combined with deionized water in a 5 to 2 mass ratio and resembled the consistency of a soft dough. At this point, the bread–water mixture was set onto the EP with the help of a plastic scaffold shown in figure 5*a* to prevent spilling throughout a series of successive drying stages. In the first stage, the PPCE and EP were dried at ambient room temperature (around 20°C) with the plastic scaffolding on for 1 hour, as shown in figure 5*b*. Once the bread mixture was set on the EP with the scaffold, it was pushed to the bottom to reach the EP. Next, the scaffolding was removed and the PPCE and EP were dried for 5 hours, as shown in figure 5*c*. Finally, the PPCE was carefully removed from the EP and was dried alone for at least 18 hours, which is shown in figure 5*d*. After these drying stages, the PPCE shown in figure 5*e* was produced with an aspect ratio of 2 absent of any evidence of apparent surface area discontinuity.

The first advantage of the reconstitution process is that the use of blending finely distributes the structure of the bread into sand-like particles. This step allows the reconstitution process to be applied to almost any recycled, dry, organic material that can be blended. For recycling purposes, discarded food may often need to be processed, cleaned or filtered before any meaningful use may be applied [45]. The viability of the reconstitution stage as a precursor to pyrolysis shows that powdery organic material can also be used to generate PPCEs, not just idealistic loaves of baked bread. Additionally, the use of blending can directly translate to an industrial recycling step, improving the fitness and scalability of the reconstitution method. Most food waste can likewise be mechanically blended or crushed into a fine powder, indicating the reconstitution process is likely applicable to other sources of biomass such as fruit peels or cereals. Other studies have also achieved pyrolysis of these now potentially recyclable materials [46]. Most notably, this process addresses the failed pattern transfer issue inherent to the stamping process. Since sufficient pattern transfer during the stamping process is observed for EPs of aspect ratios less than 2 but not equal to 2, this aspect ratio serves as a theoretical limit. A new process would be evaluated as successful by its ability to achieve pattern transfer at an aspect ratio of 2. Visual comparison between figures 5*e* and 4*d* shows that the reconstituting process outperforms the stamping process at an aspect ratio of 2 in regard to pattern transfer. A full mathematical analysis is provided later in this study.

**Figure 6.**
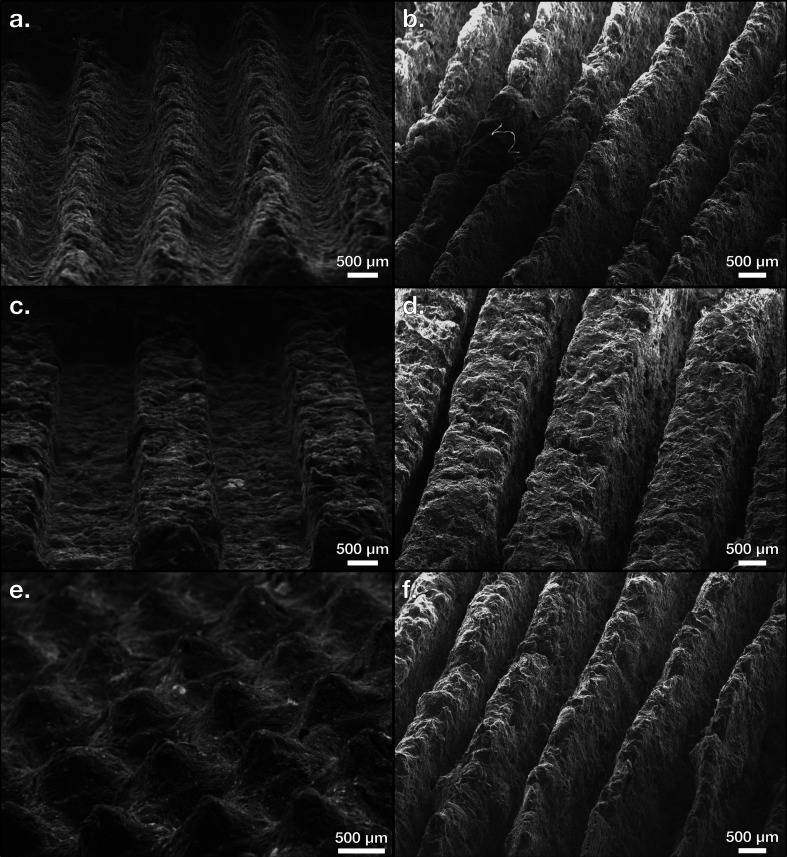
(*a*) Stamped sine wave pyrolyzed carbon electrode (PCE). (*b*) Reconstituted sine wave PCE. (*c*) Stamped square wave PCE. (*d*) Reconstituted square wave PCE. (*e*) Stamped sine matrix PCE. (*f*) Reconstituted saw wave PCE.

**Figure 7.**
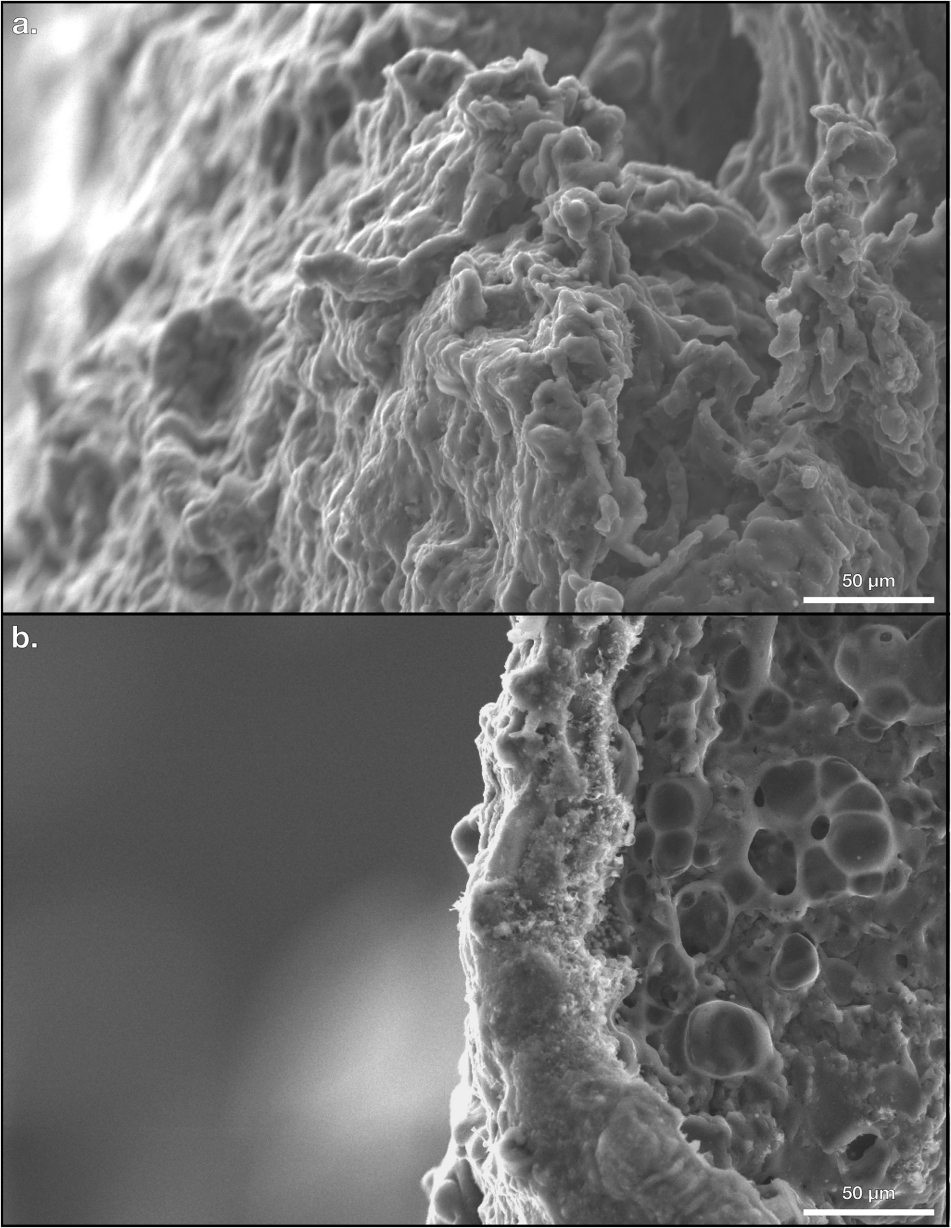
(*a*) Close-up of a stamped pyrolyzed carbon electrode (PCE). (*b*) Close-up of a reconstituted PCE.

After separating the PPCEs from the EPs of both the stamping and reconstituting methods, pyrolysis was carried out using a Lindberg Blue Tube Furnace under the flow of inert nitrogen gas at a pressure of 17500 ± 2500 kPa and a flow rate of 250 ± 50 mL/min at a temperature of 800°C for 1 hour in agreement with established protocols in literature [12]. PPCEs from both methods were loaded simultaneously into the ceramic tube, and the exhaust gas was bubbled through a beaker of water to seal the contents and monitor the flow of gas. The temperature profile was established and controlled in the tube furnace with a heating slope of 10.3°C/min and a cooling slope of 12.9°C/min to reduce the time consumed by the pyrolysis stage. Continuous purging of the interior of the tube was achieved so that thermal deformation could convert the carbon-rich PPCEs into PCEs. The shapes of both stamped and reconstituted electrodes were maintained throughout the pyrolysis stage upon release from the tube furnace. The samples were examined visually and photographed using a Hitachi S-3200N scanning electron microscope (SEM). Photos of the surface features of representative PCEs are shown in figure 6. Upon handling during laboratory transfer and storage, the final electrodes are mostly rigid, monolithic, and robust to fracture such as dropping from a height of about 1 metre. Previous studies on the production of acid-doped PCEs often lead to a porous electrode microstructure, but acidic doping was not performed in the two production pathways introduced in this work to yield a greener manufacturing method. We predict our electrodes to be stronger due to the monolithic and semi-porous structure shown in a close-up of the side profiles of each method in figure 7, though a future study on the electrodes’ mechanical stabilities is needed.

## 4. Analysis

**Figure 8.**
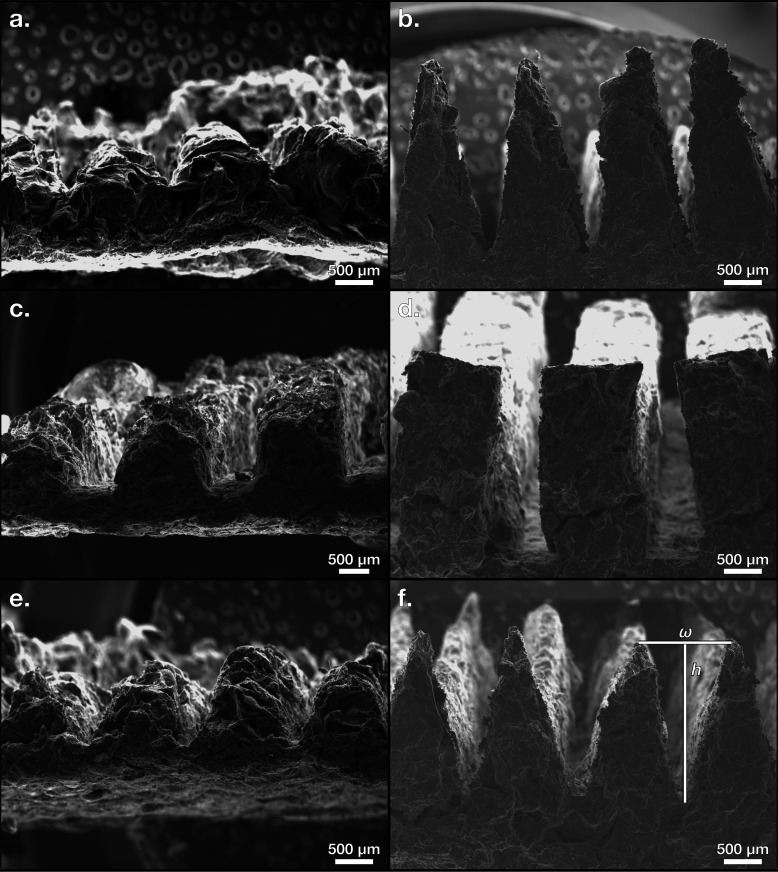
(*a*) Stamped and (*b*) reconstituted sine wave pyrolyzed carbon electrode (PCE). (*c*) Stamped and (*d*) reconstituted square wave PCE. (*e*) Stamped and (*f*) reconstituted saw wave PCE with relevant dimensions.

To quantify the surface area mating performance between the EP and PPCE of both the stamping and reconstituting methods, measurements of the parameters defined in equation (3.2) as *h* and *ω* were taken using the open-source photo analysis software ImageJ. From the two production methods, three PPCE samples of each of the three EP shapes shown in figure 2*a*–*c* were first photographed using the AmScope and Moticam setup similar to the sample shown in figure 3*d*. After the first photographing sequence, the PPCE samples were heated in the pyrolysis furnace to yield a total of 18 PCE monoliths. These 18 monoliths were photographed a second time in the SEM. Figure 8 displays the SEM photos used to measure each PCE for each production method and each EP shape. During each photographing sequence, the representative measurements shown in figure 8*f* were recorded for the same three observable features of *h* and the same three observable features of *ω* before and after pyrolysis. The key advantage of the reconstituting method over the stamping method is the fitness to retain an impressed shape. This trend is visually apparent in figure 8 as all three shapes created with the stamping method (figure 8*a*,*c*,*e*) do not demonstrate the same rigidity and resolution as the shapes created with the reconstituting method (figure 8*b*,*d*,*f*). Well-defined edge features are demanded in the precision industries of microelectronics, semiconductors, and electrode design, indicating the enhanced suitability of the reconstituted PCEs in such fields.

**Figure 9.**
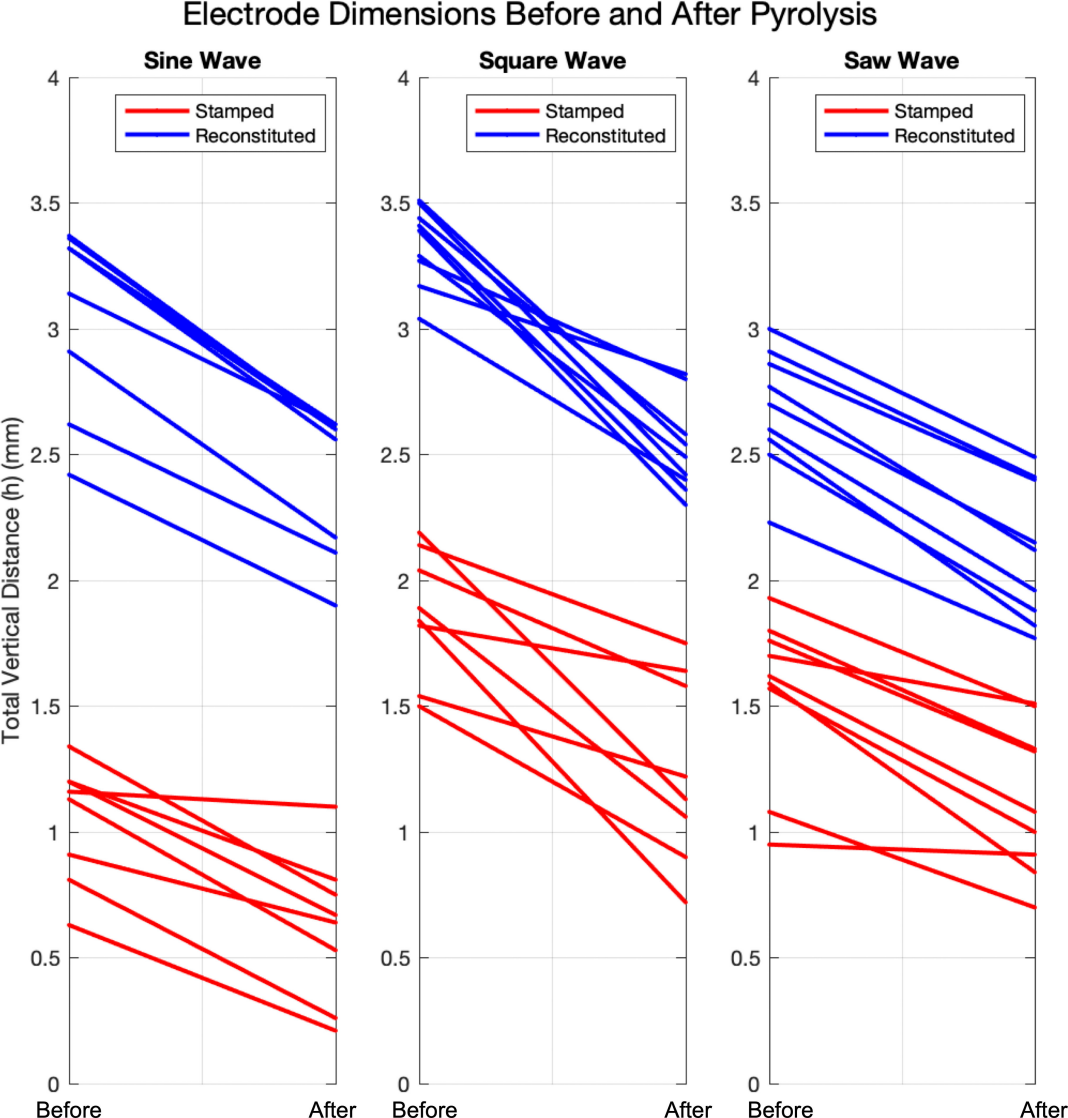
Visual summary of characteristic measurements of vertical feature distance (*h*) measured on 18 monolithic pyrolyzed carbon electrodes (PCEs) before and after pyrolysis.

Since there are two production methods, a picture of each PPCE and PCE, three EP shapes, three samples of each EP shape, and three measurements of both wavelength and height, the combined number of measurements totals to 216. For transparency, four vertical measurements of *h* are not reported due to the destruction of the features during transportation or are omitted as outliers. This leaves the number of recorded vertical measurements of *h* at 104. Figure 9 provides a visual summary of each of the 104 vertical measurements of *h* recorded for each EP shape before and after pyrolysis. Subplots are divided into each EP shape shown in figure 2*a*–*c*. The data are colour-coded according to each manufacturing process as denoted by the legend.

**Figure 10.**
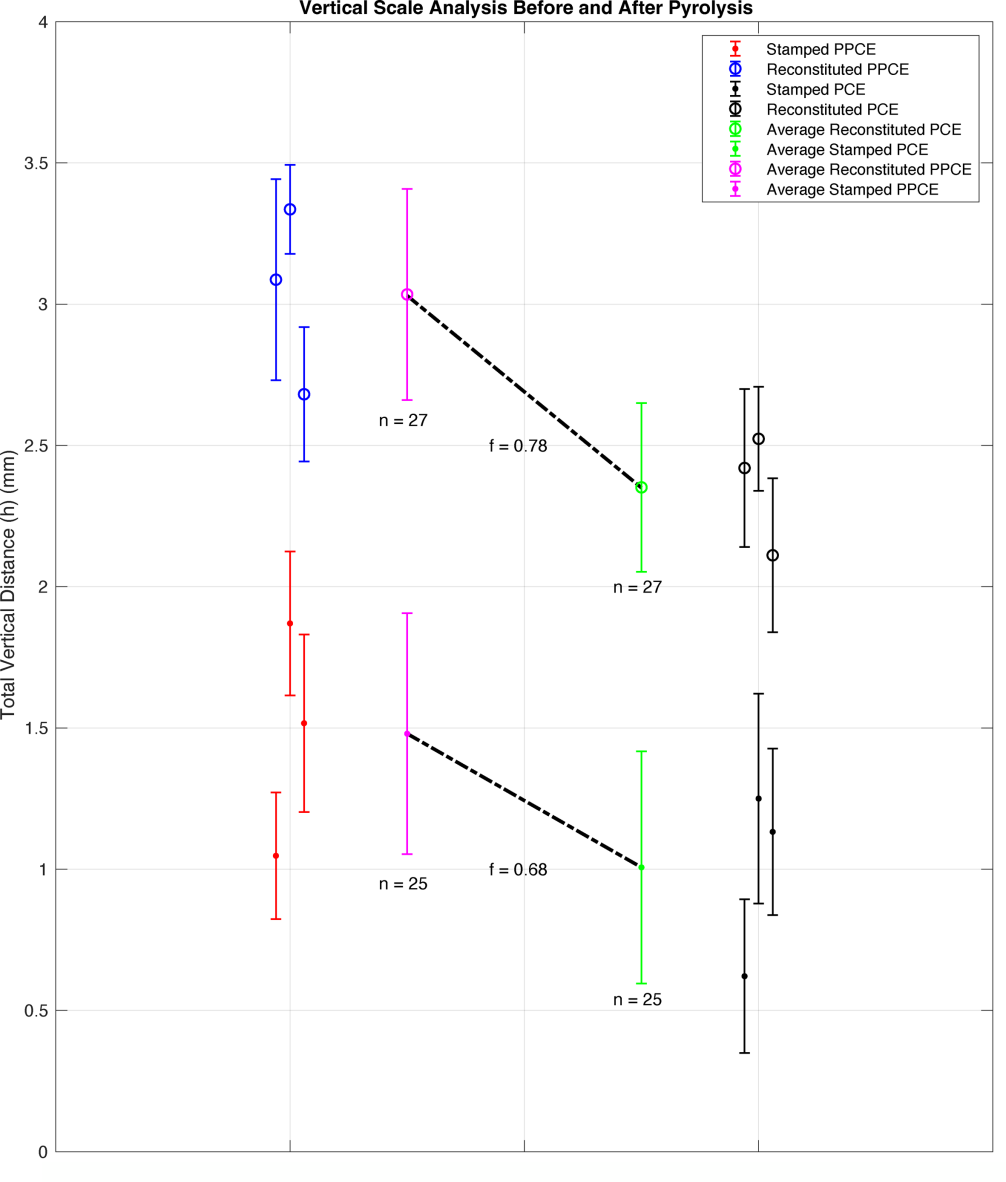
Plot of average vertical dimensions for pre-pyrolyzed carbon electrodes (PPCEs), pyrolyzed carbon electrodes (PCEs) and vertical scaling that occurs during pyrolysis of both manufacturing methods.

Now that the methodology has been provided for taking a profile of measurements of each PPCE and PCE, a method for determining the vertical scaling that occurs during pyrolysis can be obtained. From the data portrayed in subplots in figure 9, the plot shown in figure 10 documents all vertical measurements of *h* for each EP shape and each method before and after pyrolysis. Shown in blue open circles are the averages of each of the three measurements of *h* of the reconstituted PPCEs for each of the three EP shapes with error bars equal to the standard deviation. Shown in black open circles is this analysis repeated for each reconstituted PCE. Shown in closed red circles are the averages of each of the three measurements of *h* of the stamped PPCEs for each of the three EP shapes with error bars equal to the standard deviation. Shown in black closed circles is this analysis repeated for each stamped PCE.

Plotted to the left of the black sets are the average of the measurements of *h* of the reconstituted PCE set in a green open circle with error bars of standard deviation, *n* = 27, and the average of the measurements of *h* of the stamped PCE set in a green closed circle with error bars of standard deviation, *n* = 25. These points are denoted as $\overset{-}{h_{PCE}}$ and have a value of 2.35 ± 0.3 mm and 1.01 ± 0.4 mm. Plotted to the right of the blue and red sets are the average of the measurements of *h* of the reconstituted PPCE set in a pink open circle with error bars of standard deviation, *n* = 27, and the average of the measurements of *h* of the stamped PPCE set in a pink closed circle with error bars of standard deviation, *n* = 25. These points are denoted as $\overset{-}{h_{PPCE}}$ and have a value of 3.03 ± 0.4 mm and 1.48 ± 0.4 mm, respectively. The vertical scaling factor is defined in equation (4.1) for both manufacturing processes:


(4.1) $$f=\;\frac{\overset{-}{h_{PCE}}}{\overset{-}{h_{PPCE}}}.$$

A dashed line is plotted for the stamping and reconstituting methods to visualize the scaling that occurs during pyrolysis with the values of *f* for each method plotted next to the lines. The vertical scaling factor is calculated to be 0.78 for the reconstituting method and 0.68 for the stamping method.

As noted in figure 10, the series of data that are plotted with closed circles represent PPCEs and PCEs created with the stamping method while the data in open circles represent PPCEs and PCEs created with the reconstitution method. The error bars of these series are plotted as one standard deviation and do not overlap either as PPCEs or PCEs, showing that the reconstitution method is able to achieve surface area contact of vertical features between PPCEs and EPs significantly better than the stamping method.

Additionally, all three open circles of PPCEs and PCEs and all three closed circles of PPCEs and PCEs have error bars that overlap within the same dataset for both the stamping method and reconstituting method. This allows for the conclusion that the three different shapes of EPs shown in figure 2*a–c* have no observable effect on the ability to achieve full surface area contact between EP and PPCE. This means that right angles, obtuse angles, acute angles, and even curved geometries do not impact the ability of the EP to impart a shape onto the PPCE. Single-factor analysis of variance testing (ANOVA) on the magnitude of vertical distance of each PCE reaffirms the statistical difference between stamped and reconstituted PCEs, indicating that the reconstitution method produces PCEs with significantly different values of *h* than PCEs produced with the stamping method. Four data points were omitted as mentioned previously in this section for a total of 50 observations. Full statistical details are available in the electronic supplementary material.

## 5. Conclusions

The stamping manufacturing method is identified here as a non-destructive process in which an EP imparts a lasting surface deformation to a biomass substrate or PPCE that is maintained during the pyrolytic conversion phase. PCEs produced using this method are rigid and do not fracture due to their size; however, the transferrable features from an EP in the stamping method are incompatible with the aspect ratio *R* ≥ 2. To improve on the stamping manufacturing process, a second method involving the reconstitution of organic material allows for the production of PCEs in which full surface area contact is achieved for aspect ratios where *R* = 2. Both procedures are non-destructive to the EP and do not require treatment of the organic material other than mechanical agitation into a fine powder. These pathways yield sturdy and firm PCEs without the need for direct 3D printing to control the design of surface features. Since the stamping and reconstituting methods are pyrolytically indirect, several PCEs may be produced from the 3D printing of a single EP. These methods consume less time, energy, and resin since they require only one 3D printed EP, increasing their potential for incorporation into an industrial setting where the manufacturing time is limited only by the drying phase of the reconstitution method.

A greater level of surface control is obtained using the reconstitution manufacturing method, but the process shows potential areas of improvement. Though the preparation of the PPCE is not energy-intensive and requires no chemical additives, this binding process takes three successive drying phases that cumulatively last 24 hours. Working with a low-viscosity material such as remixed bread is often challenging to control, and the drying phases of the reconstitution method revealed further limitations on the resolution of the PCEs. In figure 8*b*,*d*,*f*, the PCE’s peak height seems to decrease as the peak extrudes away from the viewer and into the photograph. This phenomenon may be a result of the drying stages since the PPCE material in the centre of each peak is not directly exposed to air at times while the edges of the peaks are exposed. Despite these challenges, the two novel techniques of production proposed here have been demonstrated to yield solid monolithic PCEs, do not require the direct 3D printing of pyrolytic material, employ no chemical doping procedures, and have the capacity to incorporate recycled/processed biomaterials as an input that may otherwise be considered waste.

## Data Availability

The datasets supporting this article have been uploaded as part of the supplementary material [47].
